# Results of the first mapping of soil-transmitted helminths in Benin: Evidence of countrywide hookworm predominance

**DOI:** 10.1371/journal.pntd.0006241

**Published:** 2018-03-01

**Authors:** Moudachirou Ibikounlé, Ablavi Onzo-Aboki, Justin Doritchamou, Jean-Jacques Tougoué, Pélagie Mimonnou Boko, Boris S. Savassi, Edoux Joel Siko, Aboudou Daré, Wilfrid Batcho, Achille Massougbodji, Dorothée Akoko Kindé-Gazard, Achille Kaboré

**Affiliations:** 1 National Control Program of Communicable Diseases, Ministry of Health of Benin, Cotonou, Benin; 2 Department of Zoology, Faculty of Sciences and Techniques, University of Abomey-Calavi, Cotonou, Benin; 3 Research Triangle Institute, Washington, District of Columbia, United States of America, Research Triangle Park, NC, United States of America; 4 Department of Parasitology and Mycology, Faculty of Health Sciences, University of Abomey-Calavi, Cotonou, Benin; Yale School of Public Health, UNITED STATES

## Abstract

**Background:**

National mapping of soil-transmitted helminth infections (STH) was conducted for the first time in all of the 77 districts of Benin (West Africa) from 2013 to 2015. This mapping aimed to provide basic epidemiological data essential for the implementation of the national strategy against the neglected tropical diseases (NTDs) in the context of achieving the WHO target of controlling these infections by 2020.

**Methods:**

In each district, 5 schools were purposively selected in 5 villages and 50 school-children (25 girls and 25 boys) from ages 8 to 14 years were randomly enrolled in each school. In total, 19,250 stool samples of school children (9,625 girls and 9,625 boys) from 385 schools were examined by Kato-Katz technique.

**Results:**

The three major species of STH (hookworm, *Ascaris lumbricoides* and *Trichuris trichiura*) were observed with intra- and inter-specific variations in the prevalence and the intensity of these parasites. Hookworm infection was present in all of the surveyed districts with an average prevalence of 17.14% (95% CI 16.6%-17.6%). Among the infected schoolchildren, at national level, 90.82%, 6.73% and 2.45% of infections were of light, moderate and heavy parasite intensities respectively. A. *lumbricoides* infection, with a national average prevalence of 5.35% (95% CI 5.00%-5.60%),was the second most prevalent STH, and 84.37%, 14.27% and 1.36% of the infections were of light, moderate and heavy parasite intensities, respectively. *T*. *trichiura* had a national average prevalence of 1.15% (95% CI 0.90%-1.20%) and 80.45%, 13.18% and 6.36% infections were of light, moderate and heavy parasite intensities, respectively. The national cumulative prevalence of the three STH infections was 22.74% (95% CI 22.15%-23.33%), with58.44% (45/77) of the districts requiring mass treatment according to WHO recommendations. In all of the surveyed districts, multiple infections by STH species were common, and boys seemed more at risk of hookworm and Ascaris infections.

**Conclusions:**

This first national mapping provided an overview of the epidemiological pattern of STH infections and was essential for the implementation of a control strategy with an effective preventive chemotherapy treatment (PCT). Results show that while preventive chemotherapy is not indicated for children in 32/77 districts, 43 require annual deworming and two require twice yearly deworming. If no environmental change occurs, and no mass treatment is delivered, prevalence is likely to remain stable for many years owing to poor hygiene and sanitation.

## Author summary

Benin, like other low or moderate-income countries in the African continent, is endemic for several neglected tropical diseases, including soil-transmitted helminthiases. The National Program for Neglected Tropical Diseases of the Ministry of Health has conducted the national STH mapping using the Kato-Katz method to assess the baseline epidemiological status in all 77 districts of Benin, in order to guide implementation of a preventive chemotherapy program using albendazole. The results of the survey showed that infection with at least one of the three targeted species (hookworm, roundworm or whipworm) affected 20% or more of school aged children in 45 out of 77 districts, and which therefore require PCT. Hookworm infection was the most prevalent followed by ascariasis and trichuriasis. Boys were significantly more likely than girls to be infected with hookworm or ascariasis.

## Introduction

The soil-transmitted helminthiasis (STH) are one of eighteen groups of diseases termed as Neglected Tropical Diseases by the World Health Organization (WHO) and which have caught the attention of donors following World Health Assembly resolution WHA54.19 in 2001 [1]. Indeed, more than 1.5 billion people, or 24% of the world population, have been estimated to be infected with STH worldwide [2]. Infections are widely distributed in tropical and subtropical areas and affects the poorest and most deprived communities, with the greatest numbers occurring in sub-Saharan Africa, the Americas, China and East Asia [3,4]. These diseases are transmitted by eggs present in human excreta, which contaminate the soil in areas with populations who have limited access to latrines and potable water. The main species of STH that infect humans are nematodes (*A*. *lumbricoides*, *T*. *trichiura*, *Necator americanus* and *Ancylostoma duodenale*) with relatively similar cycles involving the presence of adult worms in the intestine. Infection is caused by larval penetration of the skin (in the case of hookworm) or ingestion of parasite eggs (for other STH). High to moderate intensity of STH infections have been associated with increased risk of malnutrition, iron-deficiency related anemia and other adverse physical and cognitive morbidities, particularly in children and pregnant women [2,5,6,7]. In sub-Saharan Africa (SSA), the majority of pregnant women are anemic (more than 60% in Benin) [8]and STH infections are among the most significant causes of anemia diagnosed during pregnancy in SSA [9].

In Benin, several studies have highlighted STH infections as a major public health problem and the related associations have been recently demonstrated on a small Beninese cohort [8–11]. Anemia had a negative impact on maternal health but also on newborn health due to significant decrease of the hemoglobin during the first months of life, leading to elevated risk of mortality and morbidity of the young child [8–11]. These studies have been the basis of sporadic preventive chemotherapy according to WHO guidelines. With the London Declaration on NTDs by 2020, the National Communicable Disease Control Program developed a strategy for the control of five NTDs in 2012 (Trachoma, Onchocerciasis and Lymphatic Filariasis, schistosomiasis and STH). Since 2013, the national plan has been funded by USAID through the ENVISION project led by Research Triangle Institute (RTI).

This study aimed to investigate the national epidemiological situation regarding the three major STH infections (hookworm, ascariasis and trichuriasis) as baseline data was essential for implementation of the PCT strategy in Benin.

## Materials and methods

### Ethical statement

This study was approved by the Comité National d’Ethique pour la Recherche en Santé (CNERS) under the authorisation reference 009/CNERS-MS from the Ministry of Health in Benin. Informed consent was obtained from the head teacher of each school and sometimes from the chief of the village, parents and teachers associations. In some districts where parents and teachers associations (PTA) exist, the head of the PTA and the head teachers received detailed explanation about the study. Individual parents were informed by the PTA and consents were secured orally. The PTA then provided a formal written approval on behalf of school children and parents. In cases where those that were to give authorisation were unable to read and write, a detailed verbal explanation of the form was given so that informed consent was obtained. Two copies of the written consent form were signed and dated. The person giving consent kept one copy and the second copy was returned to the NTD national control program. During the sampling all school children from whom no consent statement was received were replaced by other volunteers according to inclusion criteria. Participants detected with high intensity of STH or any other intestinal parasite infections were directed to healthcare centres in order to receive appropriate treatment before PCT school-based organized the following year.

### Study area

The republic of Benin is divided into 12 departments (political subdivisions), which are further divided into 77 districts. A total population is 10,008,749 inhabitants with 29.7% of total population of school age children (5–14 year old). These districts are divided into 545 sub-districts and into 3755 villages. Each sub-district has at least one unit of health and each village has at least one public school. All of the 77 districts were sampled in this study. Rainfall intensifies from the south to the north of the country. In the northern departments (Atacora, Donga, Borgou and Alibori), the annual rainfall varies between 900 mm and 1200 mm with numerous lakes and rivers feeding the region. In the southern departments (Collines, Zou, Atlantic, Littoral, Mono, Couffo Plateau and Oueme), the annual rainfall varies from 800 mm to 1200 mm [12]. The geographical location of each school surveyed, including altitude andUniversal Transverse Mercator coordinates are provided in S1 Table.

### Study design and stool collection

This study was carried out from 2013 to 2015 in all 77 districts of Benin (S1 Table). In each district, five primary schools were selected as previously described [13]. From each selected school, 50 children (25 girls and 25 boys) aged between 8 and 14 years were randomly selected. The children were given a container to provide stool samples. The containers were distributed by a team of lab technicians and the samples were collected within an hour.

### Detection of STH in stool samples

The stool samples were analyzed using the Kato-Katz method as previously described [13]. Although the Kato-Katz method has some limitations, especially in terms of sensitivity in settings with low infection intensities[14,15], this technique is the standard approach for highly endemic areas such as Benin [16]. In this study, the kits used were manufactured by Vestergaard Frandsen Group and 41.7 mg of stool was filtered through a nylon mesh and covered with cellophane previously soaked in 50% green-malachite [17]. Only one smear was prepared and examined per stool sample. The slides were observed under microscope by two technicians and their results were validated by a third slide-reader. Especially, hookworm eggs were counted from 20 to 60 minutes after the slide was preparedand other soil-transmitted helminth eggs (*A*. *lumbricoides*, *T*. *trichiura*) were counted 24 hours later. The presence of infection was recorded; the number of eggs for each parasite was tallied and the intensity of infection was reported as the number of eggs per gram of feces (epg). Egg counts were used to classify the intensity of infection into light (L), moderate (M), or heavy (H) as follows: for *A*. *duodenale / N*. *americanus* (not distinguished hookworms) 1–1,999 epg (L), 2,000–3,999 epg (M) and ≥4,000 epg (H); for *A*. *lumbricoides*, 1–4,999 epg (L), 5,000–49,999 epg (M) and ≥50,000 epg (H); for *T*. *trichiura*, 1–999 epg (L), 1,000–9,999 epg (M) and ≥10,000 epg (H) [18]. The cumulative prevalence of STH infections reflects the number of individuals infected with any one of the three STH parasites. Cases of co-infection were counted once. For quality control purposes, 10% of the slides prepared on the previous day were examined each day by an independent team of biologists.

### Data analysis

Data were double entered into Microsoft Excel 2008 (Redmond, Washington, USA). Range and consistency checks were conducted for all non-string variables. Descriptive statistics and prevalence estimates were calculated using Epi-Info 7 (CDC, Atlanta, USA) and all results with a P value of <0.05 were considered significant. The multiple comparison test chi square proportions were used to compare the prevalence by departments. The Fisher exact method of maximum likelihood and calculation of confidence intervals was used to calculate odds ratios by gender in each of the districts.

A Z-test was used to compare prevalence values between two districts and between the gender and Chi square to compare prevalence values between districts of each department. Any district with a prevalence of infection above 50% was defined as a “hotspot”of the corresponding parasite.

## Results

In total, stool samples from 19,250 school-children (9,625 boys and 9,625 girls) were screened in 385 schools of the 77 districts of the twelve departments using Kato-Katz technique. The eggs of several helminths (STH, Schistosoma and other) were found but this paper focuses only on STH infection.

### Distribution of hookworm infections

Hookworm infection was detected in all the surveyed districts with a prevalence ranging from 0.4% to 60% (Table 1). The national average prevalence of hookworm infection was 17.4% (95% CI: 16.6%-17.6%).The district of Djakotomey in the department of Couffo had the highest prevalence (60%) with hookworm infection detected in 150 children, whereas only one case was detected in the districts of Aguegue and Porto-Novo in the department of Oueme. In two districts (Toffo and Djakotomey), hookworm infection was detected in more than 50% of the surveyed children. The prevalence of hookworm infection was below 10% in 29/77 districts, and in 18 and 28 districts the prevalence ranged from 10% to 19.2% and from 20% to 43.2%, respectively. The intensity of the infection was light in most of the surveyed schools. However, moderate and heavy infections have been detected in different regions of the country with the highest prevalence of heavy infections observed in the districts of Copargo (16.67% of the infections) and Ouake (17.58%), both in the department of Djougou. In these two districts located in the northern part of the country, 33.33% (Copargo) and 27.47% (Ouake) of the hookworm infections were defined as moderate.

**Table 1:** Prevalence (%) and intensity of hookworm infections. (n: Infected invidious).

Department	District	Infected/Examined	Prevalence[95% CI]	Parasite Load		
				Light n (%)	Moderate n (%)	Heavy n (%)
				1–1999 epg	2000–3999 epg	≥4000 epg
ATACORA	COBLI	76/250	30.40[24.7–36.1]	72 (94.74)	4 (5.26)	0
	BOUKOUMBE	33/250	13.20 [9.0–17.4]	31 (93.94)	2 (6.06)	0
	MATERI	27/250	10.80 [6.9–14.6]	27 (100.00)	0	0
	KOUANDE	54/250	21.60[16.5–26.7]	52 (96.30)	2 (3.70)	0
	TOUKOUNTOUNA	11/250	4.40|1.8–6.9]	11 (100.00)	0	0
	TANGUIETA	26/250	10.40[6.6–14.1]	26 (100.00)	0	0
	NATITINGOU	15/250	6.00[3.0–8.9]	15 (100.00)	0	0
	KEROU	37/250	15.00|10.6–19.4]	25 (67.57)	12 (32.43)	0
	PEHUNCO	15/250	6.00|3.0–8.9]	10 (66.67)	5 (33.33)	0
DONGA	BASSILA	108/250	43.20[37.0–49.3]	102 (94.44)	2 (1.85)	4 (3.70)
	DJOUGOU	16/250	6.40[3.4–9.4]	16 (100.00)	0	0
	COPARGO	90/250	36.00[30.0–41.9]	45 (50.00)	30 (33.33)	15 (16.67)
	OUAKE	91/250	36.40[30.4–42.4]	50 (54.95)	25 (27.47)	16 (17.58)
BORGOU	NIKKI	18/250	7.20[4.0–10.4]	17 (94.44)	1 (5.56)	0
	PERERE	44/250	17.60[12.9–22.3]	43 (97.73)	1 (2.27)	0
	TCHAOUROU	48/250	19.20[14.3–24.1]	48 (100.00)	0	0
	PARAKOU	53/250	21.20[16.1–26.3]	52 (98.11)	1 (1.89)	0
	SINENDE	14/250	5.60[2.7–8.4]	14 (100.00)	0	0
	BEMBEREKE	15/250	6.00[3.0–8.9]	15 (100.00)	0	0
	N’DALI	57/250	23.00[17.8–28.2]	30 (52.63)	20 (35.09)	7 (12.28)
	KALALE	65/250	26.00[20.6–31.4]	32 (49.23)	25 (38.46)	8 (12.31)
ALIBORI	BANIKOARA	19/250	7.60[4.3–10.9]	19 (100.00)	0	0
	GOGOUNOU	26/250	10.40[6.6–14.2]	26 (100.00)	0	0
	MALANVILLE	7/250	2.80|0.7–4.8]	7 (100.00)	0	0
	KARIMAMA	3/250	1.20[0–2.5]	3 (100.00)	0	0
	SEGBANA	35/250	14.00|9.7–18.3]	35 (100.00)	0	0
	KANDI	12/250	4.80|2.1–7.4]	12 (100.00)	0	0
COLLINES	DASSA-ZOUME	76/250	30.40|24.7–36.1]	74 (97.36)	1 (1.32)	1 (1.32)
	OUESSE	74/250	29.60[23.9–35.2]	70 (94.60)	3 (4.05)	1 (1.35)
	SAVÈ	73/250	29.20[23.6–34.8]	67 (91.78)	1 (1.37)	5 (6.85)
	BANTE	61/250	24.40[19.1–29.7]	60 (98.36)	1 (1.64)	0
	GLAZOUE	61/250	24.40[19.1–29.7]	61 (100.00)	0	0
	SAVALOU	42/250	16.80[12.2–21.4]	36 (85.71)	6 (14.29)	0
ZOU	ABOMEY	15/250	6.00[3.0–8.9]	14 (93.33)	0	1 (6.67)
	AGBANGNIZOUN	55/250	22.00[16.9–27.1]	53 (96.36)	1 (1.82)	1 (1.82)
	BOHICON	23/250	9.20[5.6–12.8]	23 (100.00)	0	0
	COVÈ	70/250	28.00[22.4–33.6]	63 (90.00)	6 (8.57)	1 (1.43)
	DJIDJA	45/250	18.00[13.2–22.8]	43 (95.56)	2 (4.44)	0
	OUINHI	30/250	12.00[8.0–16]	29 (96.67)	1 (3.33)	0
	ZAKPOTA	58/250	23.20[18.0–28.4]	55 (94.83)	1 (1.72)	2 (3.45)
	ZOGBODOMEY	77/250	30.20[24.5–35.9]	77 (100.00)	0	0
	ZAGNANADO	24/250	9.60[5.9–13.2]	21 (87.50)	2 (8.33)	1 (4.17)
OUEME	ADJARRA	19/250	7.60[4.3–10.9]	19 (100.00)	0	0
	ADJOHOUN	33/250	13.20[9.0–17.4]	32 (96.97)	0	1 (3.03)
	AGUEGUE	1/250	0.40[0–1.2]	1 (100.00)	0	0
	AKPRO-MISSERETE	50/250	20.00[15.0–24.9]	48 (96.00)	1 (2.00)	1 (2.00)
	AVRANKOU	22/250	8.80[5.3–12.3]	22 (100.00)	0	0
	BONOU	15/250	6.00[3.0–8.9]	15 (100.00)	0	0
	DANGBO	12/250	4.80[2.1–7.4]	12 (100.00)	0	0
	PORTO-NOVO	1/250	0.40[0–1.2]	1 (100.00)	0	0
	SEME-KPODJI	14/250	5.60[2.7–8.4]	14 (100.00)	0	0
PLATEAU	ADJA OUERE	34/250	13.60[9.3–17.8]	32 (94.12)	1 (2.94)	1 (2.94)
	IFANGNI	80/250	32.00[26.2–37.8]	70 (87.50)	6 (7.50)	4 (5.00)
	POBE	66/250	26.40[20.9–31.9]	63 (95.45)	2 (3.03)	1 (1.52)
	KETOU	70/250	28.00[22.4–33.6]	64 (91.43)	5 (7.14)	1 (1.43)
	SAKÉTÉ	66/250	26.40[20.9–31.9]	63 (95.45)	3 (4.55)	0
ATLANTIQUE	ABOMEY-CALAVI	25/250	10.00[6.2–13.7]	18 (72.00)	6 (24.00)	1 (4.00)
	ALLADA	27/250	10.80[6.9–14.6]	26 (96.30)	1 (3.70)	0
	KPOMASSE	12/250	4.80[2.1–7.4]	11 (91.67)	0	1 (8.33)
	OUIDAH	24/250	9.60[5.9–13.2]	24 (100.00)	0	0
	SÔ-AVA	5/250	2.00[0.3–3.7]	5(100.00)	0	0
	TOFFO	125/250	50.00[43.8–56.2]	121 (96.80)	4 (3.20)	0
	TORI-BOSSITO	80/250	32.00[26.2–37.8]	78 (97.50)	2 (2.50)	0
	ZE	69/250	27.60[22.0–33.1]	67 (97.10)	2 (2.90)	0
LITTORAL	COTONOU	6/250	2.40[0.5–4.3]	6 (100.00)	0	0
COUFFO	APLAHOUE	81/250	32.40[26.6–38.2]	81 (100.00)	0	0
	DJAKOTOMEY	150/250	60.00[53.9–66.0]	142 (94.67)	6 (4.00)	2 (1.33)
	DOGBO	3/250	1.20[0.0–2.5]	3 (100.00)	0	0
	KLOUEKAME	96/250	38.4[32.3–44.4]	94 (97.92)	2 (2.08)	0
	LALO	73/250	29.20[23.5–34.8)	58 (79.45)	12 (16.44)	3 (4.11)
	TOVIKLIN	46/250	18.40[13.5–23.2]	45 (97.83)	1 (2.17)	0
MONO	ATHIEME	30/250	12.00[8.0–16.0]	25 (83.33)	5 (16.67)	0
	BOPA	22/250	8.80[5.3–12.3]	22 (100.00)	0	0
	COME	10/250	4.00[1.6–6.4]	9 (90.00)	0	1 (10.00)
	GRAND POPO	20/250	8.00[4.6–11.3]	18 (90.00)	2 (10.00)	0
	HOUEYOGBE	38/250	15.20[10.7–19.6]	36 (94.74)	2 (5.26)	0
	LOKOSSA	76/250	30.40[24.7–36.1]	71 (93.42)	4 (5.26)	1 (1.32)
SYNTHESIS		3300/19250	17.14[16.6–17.6]	2997 (90.82)	222 (6.73)	21 (2.45)

### Prevalence of *A*. *lumbricoides* infection

Ascariasisis less widely distributed compared to hookworm and the overall prevalence of infection was 5.35% (95% CI5.00%-5.60%). No Ascaris infection was detected in 15 districts (Table 2). In the other districts where Ascaris infection was detected, the prevalence ranged from 0.4% to 26.4%. The highest prevalence was observed in Toffo (26.4%), in the department of Atlantique in southern Benin, along with the district of Allada (21.20%) from the same department, and the districts of Bante (22.40%) and Glazoue (23.20%) both from the department of Collines. In these 4 districts, the prevalence of Ascaris infection was ≥20%. In 48/77 districts, the prevalence of Ascaris infection was <10%, whereas 10/77 districts had prevalence values between 10% and 18%. Most of the detected infections had light parasite load. All over the country, Ascaris infections with heavy parasite intensity were detected in only 6 districts, the highest being observed at Ouake (13% of infections) in the department of Djougou (Table 2). Ascaris infections with moderate parasite intensity were found in the 20 districts including the 6 districts with heavy parasite intensity.

**Table 2:** Prevalence (%) and intensity of *A*. *lumbricoides* infections.

Department	District	Infected/Examined	Prevalence [95% IC]	Parasite load		
				Light n (%)	Moderate n (%)	Heavy n (%)
				1–4999 epg	5000–49999 epg	≥50000 epg
ATACORA	COBLI	13/250	5.20[2.4–7.9]	13 (100)	0	0
	BOUKOUMBE	32/250	12.80[8.6–16.9]	32 (100)	0	0
	MATERI	32/250	12.80[8.6–16.9]	30 (93.75)	2 (6.25)	0
	KOUANDE	16/250	6.40[3.4–9.4]	15 (93.75)	1 (6.25)	0
	TOUKOUNTOUNA	15/250	6.00[3.0–8.9]	15 (100)	0	0
	TANGUIETA	12/250	4.80[2.1–7.4]	12 (100)	0	0
	NATITINGOU	0/250	0	0	0	0
	KEROU	7/250	3.00[0.9–5.1]	7 (100)	0	0
	PEHUNCO	0/250	0	0	0	0
DONGA	BASSILA	0/250	0	0	0	0
	DJOUGOU	0/250	0	0	0	0
	COPARGO	9/250	4.00[1.6–6.4]	9 (100)	0	0
	OUAKE	45/250	18.00[13.2–22.8]	30 (66.67)	9 (20.00)	6 (13.33)
BORGOU	NIKKI	0/250	0	0	0	0
	PERERE	0/250	0	0	0	0
	TCHAOUROU	14/250	5.60[2.7–8.4]	14 (100)	0	0
	PARAKOU	17/250	6.80[3.7–9.9]	17 (100)	0	0
	SINENDE	8/250	3.20[1.0–5.4]	8 (100)	0	0
	BEMBEREKE	7/250	3.00[0.9–5.1]	6 (85.7)	1 (14.3)	0
	N’DALI	0/250	0	0	0	0
	KALALE	0/250	0	0	0	0
ALIBORI	BANIKOARA	25/250	10.00[6.3–13.7]	25 (100)	0	0
	GOGOUNOU	14/250	5.60[2.7–8.4]	14 (100)	0	0
	MALANVILLE	15/250	6.00[3.0–8.9]	15 (100)	0	0
	KARIMAMA	6/250	2.40[0.5–4.3]	6 (100)	0	0
	SEGBANA	33/250	13.20[9.0–17.4]	33 (100)	0	0
	KANDI	9/250	3.60[1.3–5.9]	9 (100)	0	0
COLLINES	DASSA-ZOUME	1/250	0.40[0–1.2]	1 (100.00)	0	0
	OUESSE	0/250	0	0	0	0
	SAVÈ	3/250	1.20[0–2.5]	3 (100.00)	0	0
	BANTE	56/250	22.40[17.2–27.6]	56 (100)	0	0
	GLAZOUE	58/250	23.20[18.0–28.4]	58 (100)	0	0
	SAVALOU	11/250	4.40[1.8–6.9]	11 (100)	0	0
ZOU	ABOMEY	1/250	0.40[0–1.2]	1 (100.00)	0	0
	AGBANGNIZOUN	2/250	0.80[0–1.9]	2 (100.00)	0	0
	BOHICON	2/250	0.80[0–1.9]	2 (100.00)	0	0
	COVÈ	0/250	0	0	0	0
	DJIDJA	0/250	0	0	0	0
	OUINHI	36/250	14.40[10–18.7]	18 (50.00)	18 (50.00)	0
	ZAKPOTA	5/250	2.00[0.3–3.7]	5 (100.00)	0	0
	ZOGBODOMEY	8/250	3.20[1.0–5.4]	8 (100.00)	0	0
	ZAGNANADO	3/250	1.20[0–2.5]	3 (100.00)	0	0
OUEME	ADJARRA	6/250	2.40[0.5–4.3]	6 (100.00)	0	0
	ADJOHOUN	0/250	0	0	0	0
	AGUEGUE	12/250	4.80[2.1–7.4]	12 (100.00)	0	0
	AKPRO-MISSERETE	4/250	1.60[0.04–3.1]	4 (100.00)	0	0
	AVRANKOU	0/250	0	0	0	0
	BONOU	0/250	0	0	0	0
	DANGBO	3/250	1.20[0–2.5]	3 (100.00)	0	0
	PORTO-NOVO	1/250	0.40[0–1.2]	1 (100.00)	0	0
	SEME-KPODJI	18/250	7.20[4.0–10.4]	12 (66.67)	6 (33.33)	0
PLATEAU	ADJA OUERE	37/250	14.80[10.4–19.2]	29 (78.38)	8 (21.62)	0
	IFANGNI	18/250	7.20[4.0–10.4]	8 (44.44)	9 (50.00)	1 (5.56)
	POBE	34/250	13.60[9.3–17.8]	17 (50.00)	17 (50.00)	0
	KETOU	1/250	0.40[0–1.2]	1 (100.00)	0	0
	SAKÉTÉ	9/250	3.60[1.3–5.9]	9 (100.00)	0	0
ATLANTIQUE	ABOMEY-CALAVI	7/250	2.80[0.7–4.8]	7 (100.00)	0	0
	ALLADA	53/250	21.20[16.1–26.2]	30 (56.60)	23 (43.40)	0
	KPOMASSE	2/250	0.80[0–1.9]	2 (100.00)	0	0
	OUIDAH	4/250	1.60[0.4–3.1]	4 (100.00)	0	0
	SÔ-AVA	15/250	6.00[3.0–8.9]	10 (66.67)	5 (33.33)	0
	TOFFO	66/250	26.40[20.9–31.9]	49 (74.24)	16 (24.24)	1 (1.52)
	TORI-BOSSITO	0/250	0	0	0	0
	ZE	23/250	9.20[5.6–12.8]	19 (82.61)	4 (17.39)	0
LITTORAL	COTONOU	7/250	2.80[0.7–4.8]	7 (100.00)	0	0
COUFFO	APLAHOUE	41/250	16.40[11.8–20.9]	41 (100)	0	0
	DJAKOTOMEY	21/250	8.40[5.0–11.8]	17 (80.95)	3 (14.29)	1 (4.76)
	DOGBO	8/250	3.20[1.0–5.4]	8 (100)	0	0
	KLOUEKAME	6/250	2.40[0.5–4.3]	4 (66.67)	2 (33.33)	0
	LALO	17/250	6.80[3.7–9.9]	7 (41.18)	6 (35.29)	4 (23.53)
	TOVIKLIN	21/250	8.40[5.0–11.8]	17(80.95)	3 (14.29)	1 (4.76)
MONO	ATHIEME	4/250	1.60[0.4–3.1]	3 (75.00)	1 (25.00)	0
	BOPA	7/250	2.80[0.7–4.8]	7 (100)	0	0
	COME	39/250	15.60[11.1–20.1]	27 (69.23)	12 (30.77)	0
	GRAND POPO	1/250	0.40[0–1.2]	1 (100)	0	0
	HOUEYOGBE	13/250	5.20[2.4–7.9]	13 (100)	0	0
	LOKOSSA	17/250	6.80[3.7–9.9]	16 (94.12)	1 (5.88)	0
SYNTHESIS		1030/19250	5.35[5.0–5.6]	869 (84.37)	147 (14.27)	14 (1.36)

### *T*. *trichiura* infections in Benin

*T*. *trichiura* had the lowest prevalence at district and national level. The overall prevalence was 1.15% (Table 3). Trichuris infection was absent in 40/77 districts and most of the infections were detected at prevalence <10% in the other 37 districts. In these districts, the prevalence of Trichuris infection varied between 0.4% and 9.6%. Districts of Dassa-Zoume and Dogbo in the departments of Collines and Couffo respectively, had the highest (9.6%) prevalence of Trichuris infections. Along with these two districts, the districts of Materi in the department of Atacora, and Glazoue in the department of Collines were the only 4 districts with prevalence >5%. As for the other parasites screened in this study, the intensity of the Trichuris infections was light, whereas moderate and heavy infections were observed in 12 and 4districts, respectively. In the district of Dassa-zoume, heavy infection (41.67%; 95% CI: 35.56%-47.78%) was more prevalent as compared to moderate (20.83%: 95° CI: 15.79%-25.86%) and light (35.50%; 95% CI: 29.57%-41.43%) infections.

**Table 3:** Prevalence (%) and intensity of *T*. *trichiura* infections.

Department	District	Infected/Examined	Prevalence[95% IC]	Parasites Load		
				Light n (%)	Moderate n (%)	Heavy n (%)
				1–999 epg	1000–9999 epg	≥10000 epg
ATACORA	COBLI	0/250	0	0	0	0
	BOUKOUMBE	6/250	2.40[0.5–4.3]	6 (100)	0	0
	MATERI	23/250	9.20[5.6–12.8]	20 (86.96)	3 (13.04)	0
	KOUANDE	0/250	0	0	0	0
	TOUKOUNTOUNA	0/250	0	0	0	0
	TANGUIETA	0/250	0	0	0	0
	NATITINGOU	0/250	0	0	0	0
	KEROU	0/250	0	0	0	0
	PEHUNCO	0/250	0	0	0	0
DONGA	BASSILA	0/250	0	0	0	0
	DJOUGOU	0/250	0	0	0	0
	COPARGO	0/250	0	0	0	0
	KEROU	0/250	0	0	0	0
BORGOU	NIKKI	1/250	0.40[0–1.2]	1 (100.00)	0	0
	PÈRÈRÈ	0/250	0	0	0	0
	TCHAOUROU	8/250	3.20[1–5.4]	6 (75.00)	2 (25.00)	0
	PARAKOU	2/250	0.80[0–1.9]	2 (100)	0	0
	SINENDE	0/250	0	0	0	0
	BEMBEREKE	0/250	0	0	0	0
	N’DALI	0/250	0	0	0	0
	KALALE	0/250	0	0	0	0
ALIBORI	BANIKOARA	3/250	1.20[0–2.5]	3 (100)	0	0
	GOGOUNOU	0/250	0	0	0	0
	MALANVILLE	0/250	0	0	0	0
	KARIMAMA	0/250	0	0	0	0
	SEGBANA	4/250	1.60[0.04–3.1]	4 (100)	0	0
	KANDI	0/250	0	0	0	0
COLLINES	DASSA-ZOUMÈ	24/250	9.60[5.9–13.2]	9 (37.50)	5 (20.83)	10 (41.67)
	OUÈSSÈ	4/250	1.60[0.04–3.1]	3 (75.00)	0 (0.00)	1 (25.00)
	SAVÈ	4/250	1.60[0.04–3.1]	4 (100.00)	0	0
	BANTE	0/250	0	0	0	0
	GLAZOUE	21/250	8.40[5–11.8]	21 (100)	0	0
	SAVALOU	5/250	2.00[0.3–3.7]	5 (100)	0	0
ZOU	ABOMEY	1/250	0.40[0–1.2]	1 (100.00)	0	0
	AGBANGNIZOUN	1/250	0.40[0–1.2]	1 (100.00)	0	0
	BOHICON	3/250	1.20[0–2.5]	3 (100.00)	0	0
	COVÈ	5/250	2.00[0.3–3.7]	2 (40.00)	1 (20.00)	2 (40.00)
	DJIDJA	5/250	2.00[0.3–3.7]	2 (40.00)	2 (40.00)	1 (20.00)
	OUINHI	1/250	0.40[0–1.2]	0	1 (100.00)	0
	ZAKPOTA	10/250	4.00[1,6–6,4]	7 (70.00)	3 (30.00)	0
	ZOGBODOMEY	1/250	0.40[0–1.2]	1 (100.00)	0	0
	ZAGNANADO	3/250	1.20[0–2.5]	3 (100.00)	0	0
OUEME	ADJARRA	4/250	1.60[0.04–3.1]	4 (100.00)	0	0
	ADJOHOUN	0/250	0	0	0	0
	AGUÉGUÉ	0/250	0	0	0	0
	AKPRO-MISSÉRÉTÉ	0/250	0	0	0	0
	AVRANKOU	1/250	0.40[0–1.2]	1 (100.00)	0	0
	BONOU	1/250	0.40[0–1.2]	0	1 (100.00)	0
	DANGBO	2/250	0.80[0–1.9]	2 (100.00)	0	0
	PORTO-NOVO	3/250	1.20[0–2.5]	3 (100.00)	0	0
	SÈMÈ-KPODJI	3/250	1.20[0–2.5]	3 (100.00)	0	0
PLATEAU	ADJA OUÈRÈ	1/250	0.40[0–1.2]	1 (100.00)	0	0
	IFANGNI	2/250	0.80[0–1.9]	2 (100.00)	0	0
	POBÈ	0/250	0	0	0	0
	KÉTOU	0/250	0	0	0	0
	SAKÉTÉ	0/250	0	0	0	0
ATLANTIQUE	ABOMEY-CALAVI	0/250	0	0	0	0
	ALLADA	0/250	0	0	0	0
	KPOMASSÈ	0/250	0	0	0	0
	OUIDAH	3/250	1.20[0–2.5]	3 (100.00)	0	0
	SÔ-AVA	0/250	0	0	0	0
	TOFFO	2/250	0.80[0–01.9]	1 (50.00)	1 (50.00)	0
	TORI-BOSSITO	0/250	0	0	0	0
	ZÈ	2/250	0.80[0–01.9]	1 (50.00)	1 (50.00)	0
LITTORAL	COTONOU	10/250	4.00[1.6–6,4]	2 (20.00)	8 (80.00)	0
COUFFO	APLAHOUE	7/250	2.80[0.7–4.8]	7 (100)	0	0
	DJAKOTOMEY	0/250	0	0	0	0
	DOGBO	24/250	9.60[5.9–13.2]	22 (91.67)	1 (4.17)	0
	KLOUEKAME	0/250	0	0	0	0
	LALO	0/250	0	0	0	0
	TOVIKLIN	0/250	0	0	0	0
MONO	ATHIEME	0/250	0	0	0	0
	BOPA	0/250	0	0	0	0
	COME	12/250	4.80[2.1–7.4]	12 (100)	0	0
	GRAND POPO	0/250	0	0	0	0
	HOUEYOGBE	9/250	3.60[1.3–5.9]	9 (100)	0	0
	LOKOSSA	0/250	0	0	0	0
SYNTHESIS		221/19250	1.15[0.9–1.2]	177 (80.45)	29 (13.18)	14 (6.36)

### Soil-transmitted helminthiasis endemicity and preventive chemotherapy strategy

Cumulative prevalence of the surveyed STH parasites was analyzed by combining the determined prevalence of hookworm, Ascaris and Trichuris infections (Table 4). At least one of the three species of the targeted STH was found in all 77 surveyed districts). The average cumulative prevalence of STH infection was 22.74% (95% CI 22.15%-23.33%) and 58.44% (45/77) of the surveyed districts needed preventive chemotherapy (Table 4), as defined by WHO (STH prevalence ≥20%). Two rounds of PCT per year was needed in the districts of Toffo and Djakotomey where the detected STH prevalence was >50% (Table 4) using WHO guidelines [15]. Boys were significantly more likely to be infected compared to girls (S2 Table), with both hookworm and Ascaris, with average prevalence of 20.31% v. 14.03% (Z = 11.53; p<0.00001) and 5.79% v. 4.91% (Z = 2.68: p = 0.01), respectively. This difference was not observed with Trichuris infections. In all the surveyed districts, multiple infections by STH species was common in school age children as determined by the prevalence of coinfections with the highest number of cases (prevalence >10%)being found in the districts of Toffo (18.00%), Glazoue (16.50%), Bante and Lalo (14.40%), Djakotomey (14.00%) and Lokossa (10.80%).

**Table 4:** Cumulative STH (hookworms, Ascaris and Trichuris) prevalence and PCT strategy (n: Schoolchildren co infected by at least two STH species; schoolchildren coinfected were counted one; n1: Number of schoolchildren infected per department; PCT: Preventive chemotherapy; SAC: School age children).

Department	District	Parasitological data			PCT strategy
		Infected / Examined	STH Prevalence[95% CI]	X2 p-Value n1 (%)	
ATACORA (9)	COBLI	76/250	30.40[24.7–36.1]	X2 = 134.031 p<0.00001 453 (20.13)	1PCT/Year for SAC
	BOUKOUMBE	71/250	28.40[22.8–34.0]		1PCT/Year for SAC
	MATERI	76/250	30.40[24.7–36.1]		1PCT/Year for SAC
	KOUANDE	70/250	28.00[22.4–33.6]		1PCT/Year for SAC
	TOUKOUNTOUNA	26/250	10.40[6.6–14.2]		No PCT
	TANGUIETA	61/250	24.40[19.1–29.7]		1PCT/Year for SAC
	NATITINGOU	15/250	6.00[3.0–8.9]		No PCT
	KEROU	43/250	17.20[12.5–21.9]		No PCT
	PEHUNCO	15/250	6.00[3.0–8.9]		No PCT
DONGA (4)	BASSILA	108/250	43.20[37.0–49.3]	X2 = 86.630 p<0.00001 318 (31.80)	1PCT/Year for SAC
	DJOUGOU	21/250	8.40[5.0–11.8]		No PCT
	COPARGO	92/250	36.80[30.8–42.8]		1PCT/Year for SAC
	OUAKE	97/250	38.80[32.7–44.8]		1PCT/Year for SAC
BORGOU (8)	NIKKI	21/250	8.40[5.0–11.8]	X2 = 73.691 p<0.00001 411 (20.55)	No PCT
	PERERE	51/250	20.40[15.4–25.4]		1PCT/Year for SAC
	TCHAOUROU	71/250	28,40[22.8–34.0]		1PCT/Year for SAC
	PARAKOU	69/250	27.60[22.0–33.1]		1PCT/Year for SAC
	SINENDE	59/250	23,60[18.3–28.9]		1PCT/Year for SAC
	BEMBEREKE	18/250	7.20[4.0–10.4]		No PCT
	N’DALI	57/250	22.80[17.6–28.0]		1PCT/Year for SAC
	KALALE	65/250	26.00[20.6–31.4]		1PCT/Year for SAC
ALIBORI (6)	BANIKOARA	44/250	17.60[12.9–22.3]	X2 = 43.091 p<0.00001 313 (20.87)	No PCT
	GOGOUNOU	57/250	22.80[17.6–28.0]		1PCT/Year for SAC
	MALANVILLE	36/250	14.40[10.0–18.7]		No PCT
	KARIMAMA	33/250	13.20[9.0–17.4]		No PCT
	SEGBANA	84/250	33.60[27.7–39.4]		1PCT/Year for SAC
	KANDI	59/250	23.60[18.3–28.9]		1PCT/Year for SAC
COLLINES (6)	DASSA-ZOUME	90/250	36.00[30.0–41.9]	X2 = 32.903 p<0.00001 512 (34.13)	1PCT/Year for SAC
	OUESSE	75/250	30.00[24.3–35.7]		1PCT/Year for SAC
	SAVÈ	87/250	34.80[28.9–40.7]		1PCT/Year for SAC
	BANTE	106/250	42.40[36.3–48.5]		1PCT/Year for SAC
	GLAZOUE	101/250	40.40[34.3–46.5]		1PCT/Year for SAC
	SAVALOU	53/250	21.20[16.1–26.3]		1PCT/Year for SAC
ZOU (9)	ABOMEY	20/250	8.00[4.6–11.4]	X2 = 103.427 p<0.00001 476 (21.16)	No PCT
	AGBANGNIZOUN	57/250	22.80[17.6–28.0]		1PCT/Year for SAC
	BOHICON	31/250	12.40[8.3–16.5]		No PCT
	COVÈ	71/250	28.40[22.8–34.0]		1PCT/Year for SAC
	DJIDJA	47/250	18.80[13.9–23.6]		No PCT
	OUINHI	62/250	24.80[19.4–30.1]		1PCT/Year for SAC
	ZAKPOTA	70/250	28.00[22.4–33.6]		1PCT/Year for SAC
	ZOGBODOMEY	90/250	36.00[30.0–41.9]		1PCT/Year for SAC
	ZAGNANADO	28/250	11.20[7.3–15.1]		No PCT
OUEME (9)	ADJARRA	26/250	10.40[6.6–14.2]	X2 = 69.827 p<0.00001 221 (9.82)	No PCT
	ADJOHOUN	33/250	13.20[9.0–17.4]		No PCT
	AGUEGUE	13/250	5.20[2.4–7.9]		No PCT
	AKPRO-MISSERETE	52/250	20.80[15.8–25.8]		1PCT/Year for SAC
	AVRANKOU	23/250	9.20[5.6–12.8]		No PCT
	BONOU	15/250	6.00[3.0–8.9]		No PCT
	DANGBO	20/250	8.00[4.6–11.4]		No PCT
	PORTO-NOVO	5/250	2.00[0.3–3.7]		No PCT
	SEME-KPODJI	34/250	13.60[9.3–17.8]		No PCT
PLATEAU (5)	ADJA OUERE	72/250	28.80[23.2–34.4]	X2 = 4.171 p = 0.383 388 (31.04)	1PCT/Year for SAC
	IFANGNI	89/250	35.60[29.7–41.5]		1PCT/Year for SAC
	POBE	81/250	32.40[26.6–38.2]		1PCT/Year for SAC
	KETOU	71/250	28.40[22.8–34.0]		1PCT/Year for SAC
	SAKÉTÉ	75/250	30.00[24.3–35.7]		1PCT/Year for SAC
ATLANTIQUE (8)	ABOMEY-CALAVI	28/250	11.20[7.3–15.1]	X2 = 379.055 p<0.00001 502 (25.10)	No PCT
	ALLADA	73/250	29.20[23.6–34.8]		1PCT/Year for SAC
	KPOMASSE	14/250	5.60[2.7–8.4]		No PCT
	OUIDAH	25/250	10.00[6.3–13.7]		No PCT
	SÔ-AVA	20/250	8.00[4.6–11.4]		No PCT
	TOFFO	157/250	62.80[56.8–68.8]		2PCT/Year for SAC
	TORI-BOSSITO	82/250	32.80[27.0–38.6]		1 PCT/Year for SAC
	ZE	103/250	41.20[35.1–47.3]		1PCT/Year for SAC
LITTORAL (1)	COTONOU	8/250	3.20[1.0–5.4]	8 (3.20)	No PCT
COUFFO (6)	APLAHOUE	122/250	48.80[42.6–55.0]	X2 = 162.804 p<0.00001 525 (35.00)	1PCT/Year for SAC
	DJAKOTOMEY	150/250	60.00[53.9–66.1]		2PCT/Year for SAC
	DOGBO	32/250	12.80[8.6–16.9]		No PCT
	KLOUEKAME	87/250	34.80[28.9–40.7]		1PCT/Year for SAC
	LALO	78/250	31.20[25.4–36.9]		1PCT/Year for SAC
	TOVIKLIN	56/250	22.40[17.2–27.6]		1PCT/Year for SAC
MONO (6)	ATHIEME	31/250	12.40[8.3–16.5]	X2 = 54.397 p<0.00001 251 (16.73)	No PCT
	BOPA	25/250	10.00[6.3–13.7]		No PCT
	COME	50/250	20.00[15.0–24.9]		1PCT/Year for SAC
	GRAND POPO	23/250	9.20[5.6–12.8]		No PCT
	HOUEYOGBE	48/250	19.20[14.3–24.1]		No PCT
	LOKOSSA	74/250	30.60[24.9–36.3]		1PCT/Year for SAC
SYNTHESIS		4378/19250	22 .74[22.15–23.33]	X2 = 660.386 p<0.00001	

## Discussion

This study was part of national schistosomiasis and soil-transmitted helminthiasis mapping performed to collect baseline epidemiological data prior to launching STH MDA in Benin using WHO guidelines [18]. Results from this study provided evidence of nationwide distribution of STH parasites with variable prevalence and intensity of infection throughout the country.

Among the three STH species studied in the present work, *T*. *trichiura* had the lowest prevalence at the districts and national level. This overall low prevalence of trichuriasis confirmed data reported from previous studies in several districts of Benin [19,20]. The reported prevalence of trichuriasis in Benin, was also low as compared to other West Sub-Saharan African (WSSA) countries [7,21,22]. In contrast, ascariasis and hookworm infections are widely distributed with high prevalence in different regions of the countries.

Hookworm infections were widely distributed throughout the country and have been detected in 100% of the surveyed districts. This study clearly highlighted the predominance of hookworm infections nationwide with several hotspots where the prevalence reached 50% and more. Ascariasis was detected in 62/77 districts with the highest prevalence observed in Toffo. In this district, elevated prevalence of hookworm and Ascaris infections was detected, suggesting that populations living in that area are more vulnerable to these infections. Although, moderate to heavy hookworm and *Ascaris* infections have been observed in several districts of the country, most of the detected infections had a light parasite load. These findings confirmed that *Ascaris* and hookworm infections are endemic in all the departments of Benin and that the prevalence of STH in Benin varied steadily over localities and the current results are similar to those from our previous studies [13,19,20] and reports from other SSA countries [2,4,7,21]. Ascariasis appeared to be the predominant STH infection in the border districts between Benin and Burkina Faso (Materi and Boukoumbe), Benin and Togo (Copargo, Bante and Aplahoue), and Benin and Nigeria (Pobe, Ouinhi, Adja-Ouere and Segbana), whereas hookworm infections appeared to be predominant in peripheral districts in the northern and in the central districts in the southern part of the country. This distribution of STH infection could be explained by recently published data from Ivory Coast [22], which found a significantly positive association between STH infection rates and activities involving close contact to water and access to latrines. On the other hand, a negative association between STH infection and deworming, higher socioeconomic status, living in urban settings has been reported [22]. Our study showed that prevalence estimates in some districts are higher than those reported in previous studies from Benin [19,20], suggesting an increased exposure to STH in those districts. We believe that Benin follows the trends reported from other countries in SSA where the prevalence of the neglected tropical diseases appeared higher [7,22] than previously thought. However, this general trend of high prevalence of STH in WSSA is exceptional. In Burkina Faso for example, a recent study conducted on 3514 schoolchildren aged from 7 to 11 years randomly selected from 22 schools in 11 regions revealed low prevalence of hookworm, *A*. *lumbricoides* and *T*. *trichiura* infections [21].

In the current study the predominance of STH infections in boys compared to girls (hookworms: 21.31% of boys vs. 14.03% of girls, Z = 11.53, p<0.0001; Ascaris: 5.79% of boys vs. 4.91% of girls, Z = 2.68, p<0.01) might be explained by a difference in terms of behavior and activities [19,21].

Our study has a few limitations. First, the prevalence of STH in adult populations and particularly in pregnant women has not been investigated. Although having data on prevalence/intensity in other population groups such as pre-school-age children (PSAC) and women of child-bearing age (WCBA) is certainly useful, WHO does not strictly recommend collecting such information. However, since age prevalence curves have been well established for all three infections, information collected among school-age children can guide decision-making on treatment of this age-group, as well as of PSAC and WCBA. Data shown in this report are therefore sufficient to justify treatment in these two additional population groups as well [1].

Second, only one sample was analyzed for each participant due to the nationwide scale of this study and the limited financial and human resources. Day-to-day variation in STH detection that may influence the prevalence estimates reported in this study could not be considered [23]. Third, the impact of STH infections on the health of the Beninese population has not been addressed in the current study. Many studies conducted in Benin [8,11] and in other West Africa countries [10,24] showed that people living in the endemic areas are at risk of helminth infections, with the most vulnerable populations being pregnant women and children. Helminth and hookworm infections have been associated with poor cognitive and gross motor outcomes in infants, as well as maternal anemia in pregnant women [8,10,11,24]. The high prevalence of hookworm and its predominance in all the districts has an implication in terms of maternal child health policy, which should be strengthened.PCT for STH should be tailored to prevent sequelae and disabling consequences in these populations at risk. Those infections may cause not only maternal anemia but also affect infants' hemoglobin levels, their growth, their susceptibility to helminth infections, their cognitive development, their selective attention, their socioeconomic status, their physical fitness and their immunological responses to vaccination [25–30].

## Supporting information

S1 Table Geographical positions of the surveyed schools during the mapping in 77 districts in Benin. UTM: Universal Transverse Mercator.
(DOCX)

S2 Table Prevalence of three STH infections by sex. (DOCX)
