# Supplementary material for: Results of the first mapping of soil-transmitted helminths in Benin: Evidence of countrywide hookworm predominance
Source: PLoS Negl Trop Dis. 2018 Mar 1;12(3):e0006241. doi: 10.1371/journal.pntd.0006241 (PMC5849360; doi:10.1371/journal.pntd.0006241)
Supplement: S1 Table — UTM: Universal Transverse Mercator. (DOCX) [file pntd.0006241.s001.docx]

**S1 Table**. Geographic positions of 385 school samples

| **Department** | **District** | **Primary Public schools** | **Latitude**  **N** | **Longitude**  **E** | **Altitude (m)** |
| --- | --- | --- | --- | --- | --- |
| ATACORA | COBLI | NANAGADE | 10°28'32''28''' | 0°54'24''37''' | 229 |
|  |  | NOUAGOU | 10°30'38''72''' | 1°00'30''27''' | 270 |
|  |  | TAPOGA A | 10°34'8''25''' | 0°59'26''26''' | 228 |
|  |  | KOUNTORI | 10°24'42''61''' | 0°56'47''42''' | 205 |
|  |  | OROU KOUARE | 10°22'48''41''' | 0°59'37''60''' | 240 |
|  | BOUKOUMBE | MANTA-A | 10°20'57''85''' | 1°6'34''86''' | 242 |
|  |  | KOUTCHAGOU-A | 10°13'23''11''' | 1°6'54''38''' | 268 |
|  |  | KOUMONTCHIRGOU | 10°7'35''0''' | 1°7'58''29''' | 257 |
|  |  | KOUTCHATA-A | 10°11'26''89''' | 1°6' 12''96''' | 239 |
|  |  | KOUTAGOU | 10°8'50''3''' | 1°8'21''2''' | 272 |
|  | MATERI | NODI-A | 10°37'51''28''' | 1°1'37''53''' | 237 |
|  |  | DASSARI-A | 10°48'38''19''' | 1°8'37''43''' | 219 |
|  |  | KOUSSEGA | 10°49'38''56''' | 1°0'14''76''' | 234 |
|  |  | GOUANDE-A | 10°46'44''45''' | 0°54'58''76''' | 187 |
|  |  | PORGA A | 11°0'47''51''' | 0°59'31''61''' | 180 |
|  | KOUANDE | SEKOUGOUROU | 10°22'52''99''' | 1°45'21''28''' | 426 |
|  |  | FO TANCE | 10°24'58''0''' | 1°41'31''82''' | 434 |
|  |  | NIAROSSON | 10°11'59''22''' | 1°36'56''59''' | 484 |
|  |  | GUILMAROU | 10°33'50''20''' | 1°43'41''70''' | 461 |
|  |  | KOUBORO | 10°3'16''20''' | 1°33'13''25''' | 383 |
|  | TOUKOUNTOUNA | TOUKOUNTOUNA-C | 10°29'48''47''' | 1°22'38''80''' | 417 |
|  |  | TCHAKALAKOU | 10°32'28''36''' | 1°20'41''20''' | 438 |
|  |  | TCHAKIFAGA | 10°29'14''15''' | 1°20'46''79''' | 437 |
|  |  | NABAGA | 10°27'22''33''' | 1°22'38''23''' | 419 |
|  |  | KOUBA | 10°28'18''74''' | 1°38'24''38''' | 584 |
|  | TANGUIETA | TCHOUTCHOUBOU | 10°36'57''36''' | 1°15'48''82''' | 270 |
|  |  | MAKOU | 10°33'25''69''' | 1°9'44''71''' | 236 |
|  |  | NATAGATA | 10°33'58''98''' | 1°4'55''87''' | 238 |
|  |  | TANONGOU | 10°48'47''95''' | 1°26'10''3''' | 276 |
|  |  | BATIA | 10°53'30''33''' | 1°29'16''50''' | 259 |
| DONGA | BASSILA | KPINDI | 9°24'12''26''' | 1°25'45''37''' | 412 |
|  |  | ABILA | 8°50'55''52''' | 1°42'35''44''' | 368 |
|  |  | GBOMAKRO | 9°4'46''76''' | 1°54'22''28''' | 330 |
|  |  | KIKELE | 9°0'47''59''' | 1°43'46''02''' | 399 |
|  |  | TEROU | 9°4'20''63''' | 2°05'11''79''' | 300 |
|  | DJOUGOU | TAÏFA A | 9°41'39''86''' | 1°39'40''26''' | 454 |
|  |  | AFFON 1 | 9°44'47''34''' | 2°05'19''58''' | 346 |
|  |  | BELLEFOUGOU/A | 9°48'47''75''' | 1°43'22''09''' | 443 |
|  |  | KOLOKONDE/A | 9°53'17''57''' | 1°44'02''13''' | 437 |
|  |  | SOUBOUROUKOU/A | 9°40'04''58''' | 1°38'34''28''' | 474 |
| COLLINES | DASSA-ZOUME | AKOFODJOULE/A | 7°45'34''6''' | 2°22'12''40''' | 178 |
|  |  | DORISOME | 7°50'22''77''' | 2°16'50''65''' | 198 |
|  |  | HASSEOU | 7°33'16''25''' | 2°12'24''29''' | 139 |
|  |  | AGBAGOULE AKPAKPA | 7°53'56''12''' | 2°19'18''9''' | 170 |
|  |  | DJIBE-SOCLOGBO | 7°46'44''93''' | 2°17'44''55''' | 195 |
|  | OUESSE | ADJANA | 8°18'42''57''' | 2°24'16''81''' | 246 |
|  |  | DJADJO/A | 8°33'54''54''' | 2°14'34''67''' | 248 |
|  |  | VOSSA/A | 8°29'41''39''' | 2°21'26''81''' | 224 |
|  |  | EWONDA | 8°35'17''79''' | 2°33'6''86''' | 325 |
|  |  | AYETORO | 8°42'26''11''' | 2°45'5''45''' | 288 |
|  | SAVE | OKE-OWO/B | 8°2'55''25''' | 2°42'31''59''' | 177 |
|  |  | ATCHAKPA | 8°0'52''30''' | 2°23'34''93''' | 153 |
|  |  | OUOGHI | 8°7'25''85''' | 2°33'42''27''' | 258 |
|  |  | OLOU KABOUA-B | 8°14'36''87''' | 2°41'4''87''' | 290 |
|  |  | DJABATA | 7°53'50''74''' | 2°38'10''70''' | 187 |
|  | BANTE | BOBE | 8°25'32''42''' | 1°59'26''68''' | 278 |
|  |  | GOTCHA | 8°15'57''5''' | 1°44'16''74''' | 274 |
|  |  | PIRA-C | 8°29'40''33''' | 1°43'48''61''' | 348 |
|  |  | OLADJE-A | 8°25'3''89''' | 1°53'08''59''' | 292 |
|  |  | IDJOU | 8°5'17''39''' | 1°47'56''9''' | 219 |
|  | GLAZOUE | OUEDEME CENTRE | 8°0'47''4''' | 2°10'44''52''' | 219 |
|  |  | SOWE-B | 7°58'47''90''' | 2°9'58''69''' | 201 |
|  |  | LAGBO-B | 8°13'23''40''' | 2°11'05''98''' | 227 |
|  |  | ZAFFE CENTRE-C | 7°56'31''30''' | 2°14'14''7''' | 235 |
|  |  | EPP RIFFO A | 8°4'44''26''' | 2°19'00''41''' | 181 |
|  | SAVALOU | DOUME B | 8°25'32''42''' | 1°59'26''68''' | 278 |
|  |  | EPP AKETE | 8°15'57''5''' | 1°44'16''74''' | 274 |
|  |  | EPP LOGOZOHE B | 8°29'40''33''' | 1°43'48''61''' | 348 |
|  |  | EPP GOBADA B | - | - | 292 |
|  |  | OTTOLA B | 8°5'17''39''' | 1°47'56''9''' | 219 |
| ZOU | ABOMEY | DETOHOU | 7°11'06''20''' | 1°57'07''63''' | 192 |
|  |  | DJEGBE-HOUINLINHOUIN | 7°09'45''14''' | 2°00'38''50''' | 216 |
|  |  | GNNANSA TA | 7°14'14''84''' | 1°58'19''59''' | 177 |
|  |  | AGNANGNAN A | 7°09'50''77''' | 1°58'54''56''' | 244 |
|  |  | GBECON-ALIGOUDO/A | 7°10'14''53''' | 2°00'05''01''' | 231 |
|  | AGBANGNIZOUN | AVALI | 7°4'51''16''' | 1°57'42''11''' | 180 |
|  |  | AKODEBAKOU | 7°02'16''53''' | 1°57'12''55''' | 122 |
|  |  | HOUALA-VEKPA | 7°06'55''12''' | 1°58'19''51''' | 122 |
|  |  | SAHE-FONLI | 7°04'52''86''' | 1°56'54''35''' | 183 |
|  |  | TOKPA | 6°57'32''64''' | 2°00'03''82''' | 78 |
|  | BOHICON | MADJE | 7°09'20''86''' | 2°09'43''18''' | 109 |
|  |  | HELLOU | 7°14'14''53''' | 2°05'34''71''' | 212 |
|  |  | HOUNDOU B | 7°11'44''03''' | 2°03'20''77''' | 218 |
|  |  | FENANGNONNOU | 7°09'12''69''' | 2°04'54''00''' | 164 |
|  |  | ADAME ADATO | 7°11'18''70''' | 2°01'59''50''' | 216 |
|  | COVE | AZONNONGO | 7°19'11''23''' | 2°16'13''73''' | 92 |
|  |  | KINWEGO | 7°17'15''93''' | 2°17'04''95''' | 103 |
|  |  | NAOGON/A | 7°14'17''45''' | 2°20'15''5''' | 152 |
|  |  | DANGBEHONOU | 7°8'43''22''' | 2°21'48''4''' | 101 |
|  |  | DOVI-COGBE/A | 7°9'57''82''' | 2°20'56''56''' | 81 |
|  | DJIDJA | TOKPE | 7°27'56''30''' | 1°42'30''87''' | 198 |
|  |  | AGBOTOGBADJI | 7°26'14''48''' | 2°08'41''92''' | 141 |
|  |  | ZOUKON | 7°17'5''31''' | 2°01'21''03''' | 276 |
|  |  | AHLOKPA | 7°30'38''68''' | 2°08'03''49''' | 134 |
|  |  | LOBETA | 7°37'22''18''' | 1°52'23''98''' | 212 |
|  | OUINHI | GANGBAN | 7°00'56''29''' | 2°24'50''86''' | 28 |
|  |  | MIDJANNAGAN | 6°59'5''32''' | 2°24'39''60''' | 41 |
|  |  | TEVEDJIA/A | 7°05'47''35''' | 2°24'38''08''' | 52 |
|  |  | ADOGON | 7°02'09''35''' | 2°31'44''83''' | 55 |
|  |  | OUOKON B | 7°05'47''12''' | 2°27'44''29''' | 49 |
|  | ZAKPOTA | SOGBELANKOU | 7°17'02''22''' | 2°12'30''74''' | 156 |
|  |  | LONTONKPA | 7°13'13''03''' | 2°10'09''10''' | 163 |
|  |  | ZAKPOTA | 7°16'57''40''' | 2°09'33''26''' | 206 |
|  |  | TOGADJI | 7°20'15''01''' | 2°09'37''24''' | 151 |
|  |  | HEHOUNLI | 7°07'56''46''' | 2°17'34''65''' | 74 |
|  | ZOGBODOMEY | DON ZOUKOUTOU DJA | 6°59'58''59''' | 2°06'02''78''' | 99 |
|  |  | GOHISSSANOU | 7°01'54''28''' | 2°22'46''87''' | 46 |
|  |  | GUEME | 6° 56'0'' 93''' | 2° 4' 22'' 4''' | 80 |
|  |  | HLANHONOU | 7°03'51''70''' | 2°10'17''53''' | 57 |
|  |  | KOTOKPA | 7°05'42''99''' | 2°10'00''83''' | 102 |
|  | ZAGNANADO | AHLAN | 7°13'12''74''' | 2°28'07''95''' | 41 |
|  |  | KPOTO | 7°13'46''45''' | 2°26'37''51''' | 52 |
|  |  | AGONVE A | 7°15'13''75''' | 2°28'07''12''' | 50 |
|  |  | SAGBOVI | 7°07'36''22''' | 2°22'07''22''' | 53 |
|  |  | AKANGON | 7°01'14''57''' | 2°24'15''93''' | 39 |
| OUEME | ADJARA | DO HONGLA B | 6°28'35''19''' | 2°41'43''28''' | 49 |
|  |  | TCHAKOU A | 6°29'4''34''' | 2°42'18''21''' | 39 |
|  |  | DJAVI A | 6°30'37''98''' | 2°41'18''29''' | 42 |
|  |  | HOUEGBO B | 6°31'45''28''' | 2°40'27''65''' | 49 |
|  |  | AGBOMEY TAKPLIKPO | 6°27'18''99''' | 2°40'32''22''' | 25 |
|  | ADJOHOUN | ASSIGUI GBONGODO | 6°44'31''4''' | 2°31'32''17''' | 69 |
|  |  | AWONOU A | 6°45'20''28''' | 2°32'57''0''' | 98 |
|  |  | AWO GODO | 6°46'23''30''' | 2°32'28''18''' | 67 |
|  |  | AGONLIN | 6°39'26''84''' | 2°28'38''20''' | 26 |
|  |  | LOWE HOUENOUSSOU | 6°39'11''89''' | 2°28'25''81''' | 26 |
|  | AGUEGUE | AVAGBODJI A | 6°31'32''30''' | 2°32'21''12''' | 29,26 |
|  |  | ZOUGAME | 6°27'33''94''' | 2°32'41''96''' | 32,92 |
|  |  | HOUEDOME I | 6°28'41''50''' | 2°32'52''32''' | 37,80 |
|  |  | HOUEDOME A | 6°29'18''01''' | 2°32'53''23''' | 32,00 |
|  |  | DONOUKPA B | 6°27'45''75''' | 2°32'44''02''' | 34,74 |
|  | AKPRO-MISSERETE | GANMI A | 6°33'45''74''' | 2°36'27''96''' | 63 |
|  |  | BLEHOUAN | 6°34'01''98''' | 2°36'08''72''' | 63 |
|  |  | GBAKPO SEDJE | 6°37'9''62''' | 2°36'23''26''' | 93 |
|  |  | TOHOUIKANME | 6°36'18''12''' | 2°36'15''74''' | 80 |
|  |  | OUIYA | 6°35'16''50''' | 2°36'13''51''' | 56 |
|  | AVRANKOU | OUINDODJI B | 6°32'41''52''' | 2°36'58''73''' | 54,55 |
|  |  | GBOZOUME B | 6°34'49''66''' | 2°40'36''28''' | 64,00 |
|  |  | AGAMANDIN | 6°35'11''94''' | 2°39'47''66''' | 70,10 |
|  |  | MALE A | 6°31'11''32''' | 2°37'29''14''' | 47,24 |
|  |  | TCHAKLA OUANHO B | 6°32'02''61''' | 2°39'00''63''' | 57,30 |
|  | BONOU | DAME WOGON B | 6°56'45''21''' | 2°25'11''63''' | 42,36 |
|  |  | ATCHAONSA B | 6°51'28''06''' | 2°27'54''65''' | 74 |
|  |  | AVLANKANME | 6°56'4''01''' | 2°25'30''24''' | 40,53 |
|  |  | BONOU AYOGO | 6°54'25''89''' | 2°26'56''77''' | 42,06 |
|  |  | ASSROSSA | 6°55'9''29''' | 2°26'32''4''' | 38,70 |
|  | DANGBO | DANGBO A | 6°35'19''56''' | 2°33'5''62''' | 80,16 |
|  |  | ZOUNGUE | 6°36'54''95''' | 2°31'55''22''' | 50,59 |
|  |  | HETIN SOTA B | 6°35'16''17''' | 2°30'06''94''' | 61,26 |
|  |  | DEKIN | 6°32'54''71''' | 2°27'08''27''' | 28,65 |
|  |  | GBEKO B | 6°36'38''37''' | 2°26'57''20''' | 33,52 |
|  | PORTO-NOVO | DJASSIN HOUINVIE | 6°28'50''28''' | 2°35'29''06''' | 44,19 |
|  |  | DJASSIN | 6°28'34''29''' | 2°36'09''32''' | 38,70 |
|  |  | LOUHO A | 6°29'17''57''' | 2°35'01''91''' | 47,85 |
|  |  | LOKPODJI | 6°27'19''61''' | 2°39'19''60''' | 25 |
|  |  | ACRON C | 6°28'5''02''' | 2°37'52''74''' | 28 |
|  | SEME-KPODJI | KETONOU | 6°25'29''01''' | 2°33'22''81''' | 32 |
|  |  | GOHO | 6°26'30''55''' | 2°34'43''08''' | 29 |
|  |  | GBAKPODJI | 6°24'04''90''' | 2°29'37''16''' | 24 |
|  |  | TCHONVI-A | 6°24'21''57''' | 2°30'08''72''' | 27 |
|  |  | TORI AGONSA/A | 6°25'58''41''' | 2°35'09''21''' | 34 |
| PLATEAU | ADJA OUERE | FOUDITI | 6°53'02''73''' | 2°39'27''07''' | 125,57 |
|  |  | DAGBLA | 7°05'52''73''' | 2°36'00''41''' | 67,97 |
|  |  | HOUELI GABA | 7°04'46''22''' | 2°34'59''72''' | 71,01 |
|  |  | TOWI | 6°55'38''07''' | 2°31'03''87''' | 86,25 |
|  |  | GBANOU | 6°53'26''63''' | 2°32'52''16''' | 105,76 |
|  | IFANGNI | ITA SOUMBA | 6°41'54''55''' | 2°46'37''93''' | 89 |
|  |  | LAGBE | 6°40'57''77''' | 2°41'25''95''' | 91 |
|  |  | HOUMBO A | 6°41'45''95''' | 2°42'29''93''' | 86 |
|  |  | BANIGBE NAGOT A | 6°38'48''16''' | 2°42'24''53''' | 69 |
|  |  | DJEGOU NAGOT | 6°32'23''80''' | 2°42'38''34''' | 55 |
|  | POBE | IGBIDI IDIORO | 7°01'15''39''' | 2°40'26''44''' | 92 |
|  |  | GNANAGO | 7°12'01''19''' | 2°35'59''62''' | 89 |
|  |  | IBO EDE | 7°09'59''07''' | 2°42'16''77''' | 129 |
|  |  | OTETAN B | 7°09'25''98''' | 2°42'31''94''' | 122 |
|  |  | AHOYEYE | 6°59'19''53''' | 2°41'09''77''' | 99 |
|  | KETOU | AGONLIN KPANOU | 7°35'26''72''' | 2°29'00''40''' | 83 |
|  |  | IWOYE BENINOIS | 7°33'16''87''' | 2°44'27''58''' | 219 |
|  |  | AYEKOTONIA | 7°38'21''29''' | 2°41'15''11''' | 256 |
|  |  | IWESSOU | 7°36'32''32''' | 2°39'47''46''' | 278 |
|  |  | GBEGON | 7°16'31''24''' | 2°29'41''25''' | 55 |
|  | SAKETE | AGUIDI | 6°47'19''85''' | 2°41'07''56''' | 124,66 |
|  |  | AGADAHOUNME | 6°50'40''59''' | 2°39'27''79''' | 111,86 |
|  |  | ZIMON | 6°42'51''63''' | 2°38'17''10''' | 104,24 |
|  |  | IYOGOU TOHOU | 6°47'08''45''' | 2°35'36''22''' | 126,79 |
|  |  | DAGBLA | 6°40'47''24''' | 2°36'07''28''' | 99,66 |
| ATLANTIQUE | ABOMEY-CALAVI | DASSEKOMEY | 6°29'22''30''' | 2°15'04''52''' | 41 |
|  |  | TOGBA A | 6°27'27''56''' | 2°18'15''78''' | 37 |
|  |  | HEVIE A | 6°24'51''64''' | 2°15'49''38''' | 44 |
|  |  | ZINVIE AGOLEDJI | 6°36'13''26''' | 2°20'31''79''' | 88 |
|  |  | KPANROU | 6°40'57''36''' | 2°22'08''96''' | 30 |
|  | ALLADA | TOGOUDO A | 6°40'10''99''' | 2°10'14''08''' | 115 |
|  |  | TOKPA BOLLY | 6°42'58''08''' | 2°03'31''87''' | 52 |
|  |  | TOKPA GBEDJI | 6°43'40''06''' | 2°03'55''66''' | 96 |
|  |  | ATTOGON | 6°43'28''80''' | 2°09'58''62''' | 135 |
|  |  | AVAKPA | 6°40'20''12''' | 2°02'31''17''' | 65 |
|  | KPOMASSE | SEGBEYA | 6°30'24''01''' | 2°03'11''39''' | 62 |
|  |  | TOKPA DOME A | 6°29'50''29''' | 2°00'17''53''' | 57 |
|  |  | SEGBOHOUE A | 6°24'22''33''' | 1°57'42''92''' | 54 |
|  |  | AGONKANMEY | 6°23'30''17''' | 1°59'36''53''' | 46 |
|  |  | DEKANMEY A | 6°34'11''50''' | 1°59'46''80''' | 45 |
|  | OUIDAH | ADJARA ADOVIE/A | 6°24'37''76''' | 2°12'06''69''' | 40 |
|  |  | SEME TOHOKPA | 6°24'03''88''' | 2°11'42''99''' | 42 |
|  |  | GAKPE CENTRE | 6°25'34''51''' | 2°07'51''09''' | 42 |
|  |  | SAVI HOUEYIHO B | 6°25'35''52''' | 2°06'03''09''' | 54 |
|  |  | GBEZOUME | 6°20'31''73''' | 1°57'49''19''' | 33 |
|  | SÔ-AVA | SO ZOUNKO | 6°28'5''77''' | 2°24'54''20''' | 35 |
|  |  | AHOMEY GBLON | 6°32'14''63''' | 2°24'05''69''' | 24 |
|  |  | AHOME LOKPO | 6°34'50''41''' | 2°23'59''44''' | 33 |
|  |  | HOUEDO AGUEKON | 6°29'52''09''' | 2°27'33''16''' | 24 |
|  |  | DEKANMEY B | 6°30'31''55''' | 2°29'30''72''' | 26 |
|  | TOFFO | KPOKPA | 6°45'53''83''' | 2°04'00''33''' | 57 |
|  |  | TAKON | 6°49'06''98''' | 2°05'10''58''' | 68 |
|  |  | GBEGBE AGONSSA | 6°50'23''10''' | 2°04'48''71''' | 86 |
|  |  | HOUNGBO-DAME | 6°45'33''06''' | 2°03'05''18''' | 47 |
|  |  | TOFFO KINZOUN | 6°49'12''62''' | 2°07'31''38''' | 100 |
|  | TORI BOSSITO | AVAME A | 6°30'56''13''' | 2°12'09''18''' | 49 |
|  |  | HOUNGBO | 6°30'24''98''' | 2°11'48''38''' | 54 |
|  |  | TOGOUDO | 6°29'39''76''' | 2°08'14''82''' | 46 |
|  |  | TORI-CADA GBEDOU | 6°34'49''06''' | 2°11'22''86''' | 70 |
|  |  | DATINONKO | 6°35'07''14''' | 2°12'08''13''' | 83 |
|  | ZE | SEDJE HOUEGOUDO B | 6°44'40''65''' | 2°22'18''67''' | 35 |
|  |  | DODJI BATA | 6°41'03''83''' | 2°16'43''53''' | 117 |
|  |  | GOULO-SODJI | 6°44'11''79''' | 2°17'38''51''' | 107 |
|  |  | SESSIVAMI | 6°52'05''51''' | 2°22'51''59''' | 42 |
|  |  | AGBOHOUNSOU | 6°42'25''21''' | 2°22'05''89''' | 52 |
| LITTORAL | COTONOU | AGBATO | 6°23'40''37''' | 2°26'35''13''' | 32 |
|  |  | FIDJROSSE | 6°21'22''28''' | 2°21'41''89''' | 32 |
|  |  | YABBE A | 6°22'19''08''' | 2°28'15''78''' | 35 |
|  |  | ZOGBO | 6°23'31''40''' | 2°23'26''41''' | 35 |
|  |  | KINDONOU | 6°23'30''40''' | 2°21'41''50''' | 23 |
| COUFFO | APLAHOUE | APLAHOUE CENTRE | 6°56'26''38''' | 1°40'38''81''' | 172 |
|  |  | KISSAMEY CENTRE | 6°59'34''82''' | 1°43'51''68''' | 241 |
|  |  | DEKPO-A | 6°59'50''66''' | 1°40'48''62''' | 212 |
|  |  | GODOHOU | 7°6'17''96''' | 1°43'21''59''' | 179 |
|  |  | DAWUI | 7°1'35''79''' | 1°40'40''62''' | 215 |
|  | DJAKOTOMEY | HAGOUMI | 6°50'44''36''' | 1°45'41''84''' | 87 |
|  |  | SOKOUHOUE | 6°54'16''36''' | 1°41'03''92''' | 193 |
|  |  | KINKINHOUE | 6°55'31''41''' | 1°43'06''25''' | 178 |
|  |  | KPOBA CENTRE-A | 6°50'25''85''' | 1°39'22''97''' | 116 |
|  |  | ZOHOUDJI KPOBA | 6°49'17''75''' | 1°38'19''45''' | 111 |
|  | DOGBO | DOGBO TOTA-A | 6°48'17''70''' | 1°47'02''71''' | 98 |
|  |  | MADJRE-B | 6°49'32''36''' | 1°50'47''18''' | 104 |
|  |  | KPODAHA-A | 6°49'31''04''' | 1°42'34''66''' | 120 |
|  |  | AYOMI CENTRE-B | 6°46'34''07''' | 1°43'09''38''' | 58 |
|  |  | DEVE | 6°45'45''74''' | 1°40'00''47''' | 68 |
|  | KLOUEKAME | COUKLO1 | 6°58'45''52''' | 1°50'11''54''' | 196 |
|  |  | AHOGBEYA | 7°01'13''42''' | 1°54'04''35''' | 179 |
|  |  | EPP SAWAME | 7°05'53''86''' | 1°46'49''94''' | 204 |
|  |  | TOKAMEY ALIHO | 7°03'57''82''' | 1°49'34''3''' | 199 |
|  |  | ADJAHOUME-A | 7°02'54''55''' | 1°48'16''16''' | 235 |
|  | LALO | LOKOGBA 1 | 6°52'13''68''' | 1°52'12''95''' | 129 |
|  |  | ALLOYA | 6°51'29''76''' | 1°58'35''81''' | 69 |
|  |  | AHOMADEGBE-B | 6°52'11''99''' | 2°00'36''76''' | 47 |
|  |  | AHOUADA | 6°53'26''99''' | 1°59'05''19''' | 61 |
|  |  | KPASSAKANME | 6°54'54''81''' | 1°55'45''34''' | 91 |
|  | TOVIKLIN | ADJIDO A B | 6°55'47''15''' | 1°45'12''23''' | 151 |
|  |  | DJIGANGNONHOU | 6°54'59''16''' | 1°50'25''95''' | 186 |
|  |  | TANNOU COLA-B | 6°52'32''66''' | 1°47'25''00''' | 111 |
|  |  | HOUEDOGLI-B | 6°57'59''03''' | 1°46'41''10''' | 146 |
|  |  | DANDJEKOOHOUE | 6°54'28''75''' | 1°51'40''11''' | 155 |
|  | ATHIEME | DEDEKPOE | 6°37'27''52''' | 1°37'17''84''' | 45 |
|  |  | ATHIEME-A | 6°34'57''53''' | 1°40'06''96''' | 42 |
|  |  | ZOUNHOUE-B | 6°36'05''94''' | 1°42'06''85''' | 35 |
|  |  | KPINNOU-B | 6°35'01''81''' | 1°46'05''02''' | 37 |
|  |  | ATCHANNOU-A | 6°32'39''74''' | 1°45'22''30''' | 23 |
| MONO | HOUEYOGBE | LOBOGO-A | 6°37'23''79''' | 1°54'30''24''' | 124 |
|  |  | AGBODJI-B | 6°38'27''64''' | 1°58'52''39''' | 32 |
|  |  | GBAKPODJI-C | 6°39'56''88''' | 1°51'09''89''' | 45 |
|  |  | YEGODOE | 6°43'29''66''' | 1°52'58''81''' | 59 |
|  |  | BADAZOUIN-B | 6°34'18''87''' | 1°56'06''33''' | 63 |
|  | BOPA | AGATOGBO-D | 6°24'12''69''' | 1°55'20''78''' | 30 |
|  |  | GUEZIN KPOTA-B | 6°23'17''81''' | 1°57'01''49''' | 41 |
|  |  | BOWE GBEDJI-B | 6°27'47''85''' | 1°54'59''78''' | 64 |
|  |  | KPETOU-A | 6°25'35''46''' | 1°54'56''70''' | 21 |
|  |  | OUEDEME PEDAH 2 | 6°29'27''50''' | 1°56'38''95''' | 58 |
|  | COME | EWE CONDJI | 6°16'29''85''' | 1°47'29''70''' | 33 |
|  |  | ADJAHA | 6°19'29''89''' | 1°50'08''85''' | 37 |
|  |  | GOUNTOETO | 6°25'21''95''' | 1°48'23''56''' | 27 |
|  |  | SAGUE | 6°26'25''13''' | 1°48'14''38''' | 41 |
|  |  | ONKINHOUE | 6°16'35''14''' | 1°48'17''35''' | 13 |
|  | GRAND POPO | HOUNVI | 6°32'35''50''' | 1°51'12''69''' | 80 |
|  |  | MONHOV2 | 6°33'40''7''' | 1°49'04''52''' | 80 |
|  |  | DRE-B | 6°27'39''02''' | 1°50'09''50''' | 57 |
|  |  | TOKPA | 6°39'25''07''' | 1°49'17''65''' | 39 |
|  |  | DAYE-C | 6°30'56''37''' | 1°56'32''59''' | 72 |
|  | LOKOSSA | ZOUNGAME | 6°40'03''99''' | 1°41'39''36''' | 42 |
|  |  | URBAINE CENTRE-A | 6°37'55''54''' | 1°42'44''52''' | 62 |
|  |  | HLODO-A | 6°43'16''79''' | 1°40'4''88''' | 44 |
|  |  | HOUIN DOKODJI-A | 6°37'58''02''' | 1°45'03''44''' | 46 |
|  |  | AGNITO-A | 6°41'12''16''' | 1°47'10''82''' | 97 |
