# Supplementary material for: Results of the first mapping of soil-transmitted helminths in Benin: Evidence of countrywide hookworm predominance
Source: PLoS Negl Trop Dis. 2018 Mar 1;12(3):e0006241. doi: 10.1371/journal.pntd.0006241 (PMC5849360; doi:10.1371/journal.pntd.0006241)
Supplement: S2 Table — (DOCX) [file pntd.0006241.s002.docx]

S2 Table. Prevalence of three species of STH infections by sex

| Department | District | Prevalence by sex | | | | | |
| --- | --- | --- | --- | --- | --- | --- | --- |
|  |  | Hookworm | | *Ascaris lumbricoides* | | *Trichuris trichiura* | |
|  |  | Girls  n (%)  (N1=125) | Boys  n (%)  (N2=125) | Girls  n (%)  (N1=125) | Boys  n (%)  (N2=125) | Girls  n (%)  (N1=125) | Boys  n (%)  (N2=125) |
| ATACORA | COBLI | 30 (24.00) | 46 (36.80) | 10 (8.00) | 3 (2.40) | 0 | 0 |
|  | BOUKOUMBE | 18 (14.40) | 15 (12.00) | 17 (13.60) | 15 (12.00) | 2 (1.60) | 4 (3.20) |
|  | MATERI | 9 (7.20) | 18 (14.40) | 16 (12.80) | 16 (12.80) | 12 (9.60) | 11 (8.80) |
|  | KOUANDE | 37 (29.60) | 17 (13.60) | 6 (4.80) | 10 (8.00) | 0 | 0 |
|  | TOUKOUNTOUNA | 3 (2.40) | 8 (6.40) | 6 (4.80) | 9 (7.20) | 0 | 0 |
|  | TANGUIETA | 11 (8.80) | 15 (12.00) | 0 | 12 (9.60) | 0 | 0 |
|  | NATITINGOU | 8 (6.40) | 7 (5.60) | 0 | 0 | 0 | 0 |
|  | KEROU | 20 (16.00) | 17 (13.60) | 4 | 3 | 0 | 0 |
|  | PEHUNCO | 5 (4.00) | 15 (12.00) | 0 | 0 | 0 | 0 |
| DONGA | BASSILA | 51 (40.80) | 57 (45.60) | 0 | 0 | 0 | 0 |
|  | DJOUGOU | 5 (4.00) | 11 (8.80) | 0 | 0 | 0 | 0 |
|  | COPARGO | 50 (40.00) | 40 (32.00) | 4 | 5 | 0 | 0 |
|  | OUAKE | 46 (36.80) | 45 (50.00) | 23 | 22 | 0 | 0 |
| BORGOU | NIKKI | 6 (4.80) | 12 (9.60) | 0 | 0 | 1 (0.80) | 0 |
|  | PERERE | 12 (9.60) | 32 (25.60) | 0 | 0 | 0 | 0 |
|  | TCHAOUROU | 19 (15.20) | 29 (23.20) | 8 (6.40) | 6 (4.80) | 2 (1.60) | 6 (4.80) |
|  | PARAKOU | 16 (12.80) | 37 (29.60) | 7 (5.60) | 10 (8.00) | 2 (1.60) | 0 |
|  | SINENDE | 3 (2.40) | 11 (8.80) | 5 (4.00) | 3 (2.40) | 0 | 0 |
|  | BEMBEREKE | 7 (5.60) | 8 (6.40) | 3 | 4 | 0 | 0 |
|  | N’DALI | 20 (16.00) | 37(29.60) | 0 | 0 | 0 | 0 |
|  | KALALE | 28 (22.40) | 37 (29.60) | 0 | 0 | 0 | 0 |
| ALIBORI | BANIKOARA | 9 (7.20) | 10 (8.00) | 13 (10.40) | 12 (9.60) | 1 (0.80) | 2 (1.60) |
|  | GOGOUNOU | 15 (12.00) | 11 (8.80) | 7 (5.60) | 7 (5.60) | 0 | 0 |
|  | MALANVILLE | 3 (2.40) | 4 (3.20) | 4 (3.20) | 2 (1.60) | 0 | 0 |
|  | KARIMAMA | 1 (0.80) | 2 (1.60) | 5 (4.00) | 15 (12.00) | 0 | 0 |
|  | SEGBANA | 17 (13.60) | 18 (14.40) | 20 (16.00) | 13 (10.40) | 4 (3.20) | 0 |
|  | KANDI | 5 (4.00) | 7 (5.60) | 2 (1.60) | 7 (5.60) | 0 | 0 |
| COLLINES | DASSA-ZOUME | 39 (31.20) | 37 (29.60) | 1 (0.80) | 0 | 12 (9.60) | 12 (9.60) |
|  | OUESSE | 31 (24.80) | 43 (34.40) | 0 | 0 | 1 (0.80) | 3 (2.40) |
|  | SAVÈ | 23 (18.40) | 50 (40.00) | 3 (2.40) | 0 | 3 (2.40) | 1 (0.80) |
|  | BANTE | 16 (12.80) | 45 (36.00) | 21 (16.80) | 35 (28.00) | 0 | 0 |
|  | GLAZOUE | 27 (21.60) | 34 (27.20) | 29 (23.20) | 19 (15.20) | 15 (12.00) | 6 (4.80) |
|  | SAVALOU | 18 (14.40) | 24 (19.20) | 5 (4.00) | 6 (4.80) | 5 (4.00) | 0 |
| ZOU | **ABOMEY** | 6 (4.80) | 9 (7.20) | 0 | 1 (0.80) | 1 (0.80) | 0 |
|  | **AGBANGNIZOUN** | 19 (15.20) | 36 (28.80) | 1 (0.80) | 1 (0.80) | 1 (0.80) | 0 |
|  | **BOHICON** | 11 (8.80) | 12 (9.60) | 2 (1.60) | 1 (0.80) | 1 (0.80) | 2 (1.60) |
|  | **COVÈ** | 31 (24.80) | 39 (31.20) | 0 | 0 | 1 (0.80) | 4 (3.20) |
|  | **DJIDJA** | 17 (13.60) | 28 (22.40) | 0 | 0 | 2 (1.60) | 3 (2.40) |
|  | **OUINHI** | 11 (8.80) | 19 (15.20) | 18 (14.40) | 18 (14.40) | 1 (0.80) | 0 |
|  | **ZAKPOTA** | 25 (20.00) | 33 (26.40) | 2 (1.60) | 3 (2.40) | 4 (3.20) | 6 (4.80) |
|  | **ZOGBODOMEY** | 34 (27.20) | 43 (34.40) | 3 (2.40) | 5 (4.00) | 1 (0.80) | 0 |
|  | **ZAGNANADO** | 8 (6.40) | 16 (12.80) | 1 (0.80) | 2 (1.60) | 0 | 3 (2.40) |
| OUEME | **ADJARRA** | 3 (2.40) | 16 (12.80) | 4 (3.20) | 2 (1.60) | 1 (0.80) | 3 (2.40) |
|  | **ADJOHOUN** | 12 (9.60) | 21 (16.80) | 0 | 0 | 0 | 0 |
|  | **AGUEGUE** | 0 | 1 (0.80) | 6 (4.80) | 6 (4.80) | 0 | 0 |
|  | **AKPRO-MISSERETE** | 15 (12.00) | 35 (28.00) | 0 | 4 (3.20) | 0 | 0 |
|  | **AVRANKOU** | 7 (5.60) | 15 (12.00) | 0 | 0 | 1 (0.80) | 0 |
|  | **BONOU** | 2 (1,60) | 13 (10.40) | 0 | 0 | 1 (0.80) | 0 |
|  | **DANGBO** | 4 (3.20) | 8 (6.40) | 1 (0.80) | 2 (1.60) | 1 (0.80) | 1 (0.80) |
|  | **PORTO-NOVO** | 1 (0.80) | 0 | 1 (0.80) | 0 | 1 (0.80) | 2 (1.60) |
|  | **SEME-KPODJI** | 8 (6.40) | 6 (4.80) | 6 (4.80) | 12 (9.60) | 1 (0.80) | 2 (1.60) |
| PLATEAU | **ADJA OUERE** | 11 (8.80) | 23 (18.40) | 16 (12.80) | 21 (16.80) | 1 (0.80) | 0 |
|  | **IFANGNI** | 31 (24.80) | 49 (39.20) | 9 (7.20) | 9 (7.20) | 1 (0.80) | 1 (0.80) |
|  | **POBE** | 24 (19.20) | 42 (33.60) | 10 (8.00) | 24 (19.20) | 0 | 0 |
|  | **KETOU** | 23 (18.40) | 47 (37.60) | 0 | 1 (0.80) | 0 | 0 |
|  | **SAKÉTÉ** | 17 (13.60) | 49 (39.20) | 5 (4.00) | 4 (3.20) | 0 | 0 |
| ATLANTIQUE | **ABOMEY-CALAVI** | 7 (5.60) | 18 (14.40) | 1 (0.80) | 6 (4.80) | 0 | 0 |
|  | **ALLADA** | 9 (7.20) | 18 (14.40) | 27 (21.60) | 26 (20.80) | 0 | 0 |
|  | **KPOMASSE** | 6 (4.80) | 6 (4.80) | 1 (0.80) | 1 (0.80) | 0 | 0 |
|  | **OUIDAH** | 9 (7.20) | 15 (12.00) | 1 (0.80) | 3 (2.40) | 1 (0.80) | 2 (1.60) |
|  | **SÔ-AVA** | 2 (1,60) | 3 (2.40) | 9 (7.20) | 6 (4.80) | 0 | 0 |
|  | **TOFFO** | 51 (40.80) | 74 (59.20) | 30 (24.00) | 36 (28.80) | 1 (0.80) | 1 (0.80) |
|  | **TORI-BOSSITO** | 37 (29.60) | 43 (34.40) | 0 | 0 | 0 | 0 |
|  | **ZE** | 28 (22.40) | 41 (32.80) | 11 (8.80) | 12 (9.60) | 2 (1.60) | 0 |
| LITTORAL | **COTONOU** | 4 (3.20) | 2 (1,60) | 0 | 7 (5.60) | 2 (1.60) | 8 (6.40) |
| COUFFO | APLAHOUE | 30 (24.00) | 51 (40.80) | 14 (11.20) | 27 (21.60) | 7 (5.60) | 0 |
|  | DJAKOTOMEY | 73 (58.40) | 77(61.60) | 8 (6.40) | 13 (10.40) | 0 | 0 |
|  | DOGBO | 1 (0.80) | 2 (1.60) | 3 (2.40) | 5 (4.00) | 12 (9.60) | 12 (9.60) |
|  | KLOUEKAME | 44 (35.20) | 52 (41.60) | 3 (2.40) | 3 (2.40) | 0 | 0 |
|  | LALO | 22 (17.60) | 51 (40.80) | 10 (8.00) | 7 (5.60) | 0 | 0 |
|  | TOVIKLIN | 25 (20.00) | 21 (16.80) | 7 (5.60) | 14 (11.20) | 0 | 0 |
| MONO | ATHIEME | 15 (12.00) | 15 (12.00) | 3 (2.40) | 1 (0.80) | 0 | 0 |
|  | BOPA | 10 (8.00) | 12 (9.60) | 4 (3.20) | 3 (2.40) | 0 | 0 |
|  | COME | 1 (0.80) | 9 (7.20) | 18 (14.40) | 21 (16.80) | 2 (1.60) | 10 (8.00) |
|  | GRAND POPO | 9 (7.20) | 11 (8.80) | 1 (0.80) | 4 (3.20) | 0 | 0 |
|  | HOUEYOGBE | 14 (11.20) | 24 (19.20) | 9 (7.20) | 4 (3.20) | 7 (5.60) | 2 (1.60) |
|  | LOKOSSA | 25 (20.00) | 51(40.80) | 9 (7.20) | 8 (6.40) | 0 | 0 |
| SYNTHESIS |  | 1350 (14.03) | 1955 (20.31) | 473 (4.91) | 557 (5.79) | 114 (1.18) | 107 (1.11) |
